# Exploiting Unsupervised Free-Living Data for Cardiorespiratory Fitness Estimation: Systematic Review and Meta-Analysis

**DOI:** 10.2196/69996

**Published:** 2026-01-27

**Authors:** Alexios Dosis, Aron Berger Syversen, Mikolaj R Kowal, Daniel Grant, Jim Tiernan, David Wong, David G Jayne

**Affiliations:** 1 Leeds Institute of Medical Research, University of Leeds, Clinical Sciences Building, St James's University Hospital, Beckett Street, Leeds, LS97LN, United Kingdom, 44 07754226212; 2 Abdominal Medicine and Surgery, Leeds Teaching Hospitals, Leeds, United Kingdom; 3 School of Computing, University of Leeds, Leeds, England, United Kingdom; 4 Cardiorespiratory Department, Leeds Teaching Hospitals, Leeds, United Kingdom; 5 5 Leeds Institute of Health Sciences, University of Leeds, Leeds, England, United Kingdom

**Keywords:** wearables, cardiorespiratory fitness, free-living data, machine learning, perioperative medicine

## Abstract

**Background:**

Current methods of cardiorespiratory fitness (CRF) assessment may discriminate against frail individuals who are challenged to perform a maximal cardiopulmonary exercise test. CRF estimations from free-living wearable data, captured over extended time periods, may offer a more representative assessment and increase usability in clinical settings.

**Objective:**

This study aimed to review current evidence behind this novel concept and evaluate the performance and quality of models developed to estimate CRF from free-living, unsupervised data.

**Methods:**

Following the PRISMA (Preferred Reporting Items for Systematic Reviews and Meta-Analyses) guidelines, we systematically searched 4 databases (MEDLINE, Embase, Scopus, and arXiv) for studies reporting the development of models to estimate CRF from continuous free-living wearable data. Studies conducted entirely under controlled laboratory conditions were excluded. Performance metrics were combined in a meta-correlation analysis using a random-effects model and Fisher Z transformation.

**Results:**

Of 1848 papers screened, 18 met the eligibility criteria, with a total of 31,072 participants. The weighted mean age was 46.9 (SD 1.46) years. Multiple computational techniques were used, with 8 studies employing more advanced machine learning models. The meta-correlation analysis revealed a pooled overall estimate of 0.83 with a 95% CI 0.77‐0.88. The *I*^2^ test indicated high heterogeneity at 97%. Risk of bias assessment found most concerns in the data analysis domain, with studies often lacking clarity around the data handling process.

**Conclusions:**

A promising preliminary agreement between CRF predictions and measured values was noted. However, no definite conclusions can be drawn for clinical implementation due to high heterogeneity among the included studies and lack of external validation. Nonetheless, continuous data streams appear to be a valuable resource that could lead to a step change in how we measure and monitor CRF.

## Introduction

Cardiorespiratory fitness (CRF) is regarded as a key element of anesthetic preassessment and preoperative decision-making, reflecting an individual’s aerobic capacity and ability to withstand and recover from surgery. The most widely recognized measure of CRF is maximal oxygen uptake (VO_2_max), a strong indicator of the cardiorespiratory system’s ability to capture, transport, and use oxygen during exercise. VO_2_max is inversely related to all-cause mortality and linked to several other health outcomes, such as cardiovascular disease (CVD), dementia, and depression [1-3]. In surgery, VO_2_max serves as a prognostic marker and is currently the gold standard predictor of early postoperative cardiorespiratory morbidity [4,5].

Cardiopulmonary exercise testing (CPET) is a maximal dynamic test used to assess CRF and global exercise response. CPET is typically performed on a cycle ergometer or a treadmill under conditions of graduated physiological stress, involving computerized gas-exchange analysis of breath-by-breath ventilation. The test is routinely used in cardiorespiratory medicine as a diagnostic tool to distinguish between ventilatory and cardiac exercise intolerance [6]. In the preoperative setting, it is usually used as a risk assessment tool for major surgery to aid clinical decision-making regarding suitability for surgery and to guide perioperative management [7,8].

Despite its proven ability for risk stratification, there remain some drawbacks to this method. VO_2_max measurements through maximal exercise can be strenuous and challenging for older or frail adults and those with musculoskeletal conditions who may be limited by pain rather than exertion [9]. Performance-reducing factors, such as peripheral arterial disease, osteoarthritis, and poor effort, have also been associated with inaccurate measurements, which may impact clinical decision-making [10]. In addition, high costs, the requirement for highly trained staff to undertake the test, and reduced hospital availability render regular VO_2_max monitoring impractical.

To overcome these limitations, several VO_2_max prediction models have been developed: nonexercise models are usually derived from lifestyle and anthropometric data, while submaximal exercise tests rely on prespeciﬁed protocols that involve heart rate (HR) monitoring at certain speeds, such as the 20-meter shuttle test or the modified shuttle walking test [9,11,12]. These methods offer an alternative; yet, they are not widely used in routine clinical practice due to some inherent limitations. Submaximal tests rely on the assumption that mechanical efficiency is the same for everyone, often leading to inaccurate VO_₂_max estimations, and self-reported physical activity measures are subject to social desirability and recall bias [13]. Equally, lack of protocol standardization has raised concerns about the validity and reliability of submaximal tests [14]. Nonetheless, it seems a great limitation of the above CPET alternatives is their inability to capture and assess unstructured and incidental ambulatory activity accurately [15].

This gap has encouraged the exploration of wearable devices that can collect a substantial amount of information about an individual’s activities in daily life, regardless of frequency, duration, or intensity [16]. Wearable technology has experienced a remarkable uptake over the last years, with more users appreciating the potential benefits for health and fitness tracking [17]. Commercially available wearables already offer VO_2_max estimations; however, their algorithms are primarily based on short periods of structured exercise data, and their resulting VO_2_max estimations have a large degree of error at the individual level [18]. Continuous monitoring of unstructured physical activity, however, shows promise in a variety of settings, enabling constant tracking of physiological data in an unobtrusive manner [16]. Physiological signals captured over longer periods may be more representative of CRF for certain populations. In view of the great potential of these devices, we aimed to explore whether CRF can be accurately predicted leveraging wearable data from unsupervised free-living conditions, outside the controlled laboratory environment. In this paper, we systematically review the research methodology behind the proposed models, the associated challenges and limitations, and discuss the feasibility of applying this concept for CRF estimation in health care settings.

## Methods

### Search Strategy and Study Selection Process

This study was registered with the international database for systematic reviews PROSPERO (CRD42024593878). We followed the PRISMA (Preferred Reporting Items for Systematic Reviews and Meta-Analyses) statement and recommendations for systematic reviews [19]. Relevant studies were located from a systematic electronic search of 4 databases (MEDLINE, Embase, Scopus, and arXiv), and the last search was performed on July 27, 2024. The full search strategy and key terms are available in Multimedia Appendix 1.

We used online systematic review software to blind reviewers and screen titles and abstracts after removing duplicates [20]. Conflicts were resolved through direct discussion between the 2 reviewers (AD and ABS) after unblinding. The full papers of potentially eligible studies were scrutinized against the eligibility criteria. Citation chaining of references was also completed by AD. Any additional studies identified were subsequently reviewed and assessed for inclusion by a third investigator (MRK; Textbox 1).

Textbox 1. **Inclusion and exclusion criteria.**

**Inclusion criteria:**

- All papers reporting the development of a prediction model to estimate maximal oxygen uptake from longitudinal free-living wearable data.
- “Free-living” is defined as data collected in unsupervised, uncontrolled, real-world settings.
- Mixed designs with some simulated activities permitted, provided that at least some unsupervised activity was captured and analyzed.
- Human studies published in English.
- All wearable devices are eligible, including accelerometers, electrocardiogram (ECG) biosensors, commercial smartwatches, and optical heart rate sensors (photoplethysmogram).
- No restriction applied to the clinical setting studied.

**Exclusion criteria:**

- Studies in which the authors focused solely on physical activity and energy expenditure estimation.
- Monitoring occurring exclusively under controlled laboratory conditions, with no free-living activity studied.
- Wearable data including only exercise activity.
- Studies in which the authors did not report a prediction model but only correlations of wearable metrics with measures of cardiorespiratory fitness.
- Systematic reviews, literature reviews, surveys, conference proceedings, or meeting proceedings.
- Studies focusing on adolescents and young children.

We considered all papers reporting the development of a prediction model to estimate VO_2_max from longitudinal free-living wearable data. For this review, “free-living” was defined as data collected in unsupervised, uncontrolled, real-world settings. Mixed designs with some simulated activities were also permitted, provided that at least some unsupervised activity was being captured and analyzed by the authors. A limit was set to human studies published in English. All wearable devices were eligible, including accelerometers, electrocardiogram (ECG) biosensors, commercial smartwatches, and optical HR sensors (photoplethysmogram). No restriction was applied to the clinical setting studied.

Studies were disqualified if (1) the authors focused solely on physical activity and energy expenditure estimation; (2) monitoring occurred exclusively under controlled conditions in a laboratory setting, and no free-living activity was studied; (3) wearable data included only exercise activity; (4) the authors did not report a prediction model, but only correlations of various wearable metrics with measures of CRF; (5) they were systematic or literature reviews, surveys, conference, or meeting proceedings; and (6) studies focusing on adolescents and young children.

### Data Extraction and Model Performance Assessment

To ensure consistency, a standardized form was piloted and modified until consensus was reached between 2 authors and the senior investigator for the data extraction tool. Two reviewers (AD and ABS) retrieved all relevant data independently, and a third author (MRK) verified the accuracy of the records, cross-referencing with sources to resolve discrepancies. For each study, the following items were extracted: study details, demographics, setting and sample size, wearable device used for monitoring, and the baseline method used to obtain the ground truth (control). We recorded features derived from wearable data and the preprocessing techniques researchers used for feature extraction. We extracted details on the various machine learning (ML) models that were used, as well as prediction accuracy metrics and the validation process reported.

### Quality Assessment

Qualitative appraisal of each included study was independently performed by 2 authors using a modified version of the Prediction model Risk Of Bias Assessment Tool (PROBAST) following the updated TRIPOD-AI (Transparent Reporting of a multivariable or ML prediction model for Individual Prognosis Or Diagnosis–artificial intelligence) guidance [21,22]. In case of disagreements, the opinion of the third author was sought.

Studies received a score of “low,” “unclear,” or “high” risk of bias on five major domains: (1) predictor choice and definition; (2) participant selection, including source and study setting; (3) outcome measurement; and (4) analysis and methodological quality of the proposed model. Overall judgment was rated as unclear if at least 1 domain was regarded as unclear, and similarly as high if any domain was rated as high. Risk-of-bias plots were created for quality assessment using the “robvis” software package in R (R Foundation for Statistical Computing).

### Data Analysis

We calculated and reported descriptive statistics to outline each study characteristic. Summary measures are reported as means or medians, including measures of dispersion such as SDs. Key metrics of model accuracy were identified and combined for quantitative analysis.

Frequently reported metrics included the Pearson correlation coefficient (*r*) and the *R*^2^ values. Other metrics such as the standard error of estimate (SEE) and the root-mean-square error were also reported but less frequently. Although not technically an accuracy metric, where available, the *r* coefficient was used to indicate how well the model’s predictions aligned with the CPET values.

A meta-analysis of correlation estimates was undertaken to integrate measures of performance across the included studies and provide a more objective and systematic assessment. In this instance, reported correlation coefficients (*r*) were used as the primary effect size, as they represented the most consistently reported metric in this review. When only *R*² values were available, we converted them by taking the square root to obtain the corresponding *r* values between the predicted and actual VO_2_max values and assessing the direction of association in each study. RStudio (version 2024.04.2+764; R Foundation for Statistical Computing) was used for all statistical analyses [23]. Two packages, “metafor” and “robumeta,” were installed to perform the meta-analysis. Fisher Z transformation was applied to convert correlation estimates to a more normally distributed metric and obtain standard effect sizes. A restricted maximum likelihood estimation method was used for a standard random-effects model to conduct the meta-analysis [24]. The random-effects model assigns less study weight to larger studies with less variance [24]. Results of the meta-correlation analysis were presented visually using a forest plot. Subgroup analysis was also performed, comparing regression-based models with more advanced ML methodologies.

We assessed heterogeneity using the *I*^2^ and τ^2^ statistics. The *I*^2^ statistic quantifies the proportion of total variation in effect sizes that was due to heterogeneity rather than chance. We considered *I*^2^ values of 25%, 50%, and 75% to represent low, moderate, and high heterogeneity, respectively [25]. The τ^2^ represents another method to assess the between-study variance, focusing on the absolute variability of true effect size, with higher values indicating greater heterogeneity [26]. The Egger test was used to assess the likelihood of publication bias, which was also presented visually with a funnel plot.

## Results

### Overview

**Figure 1.**
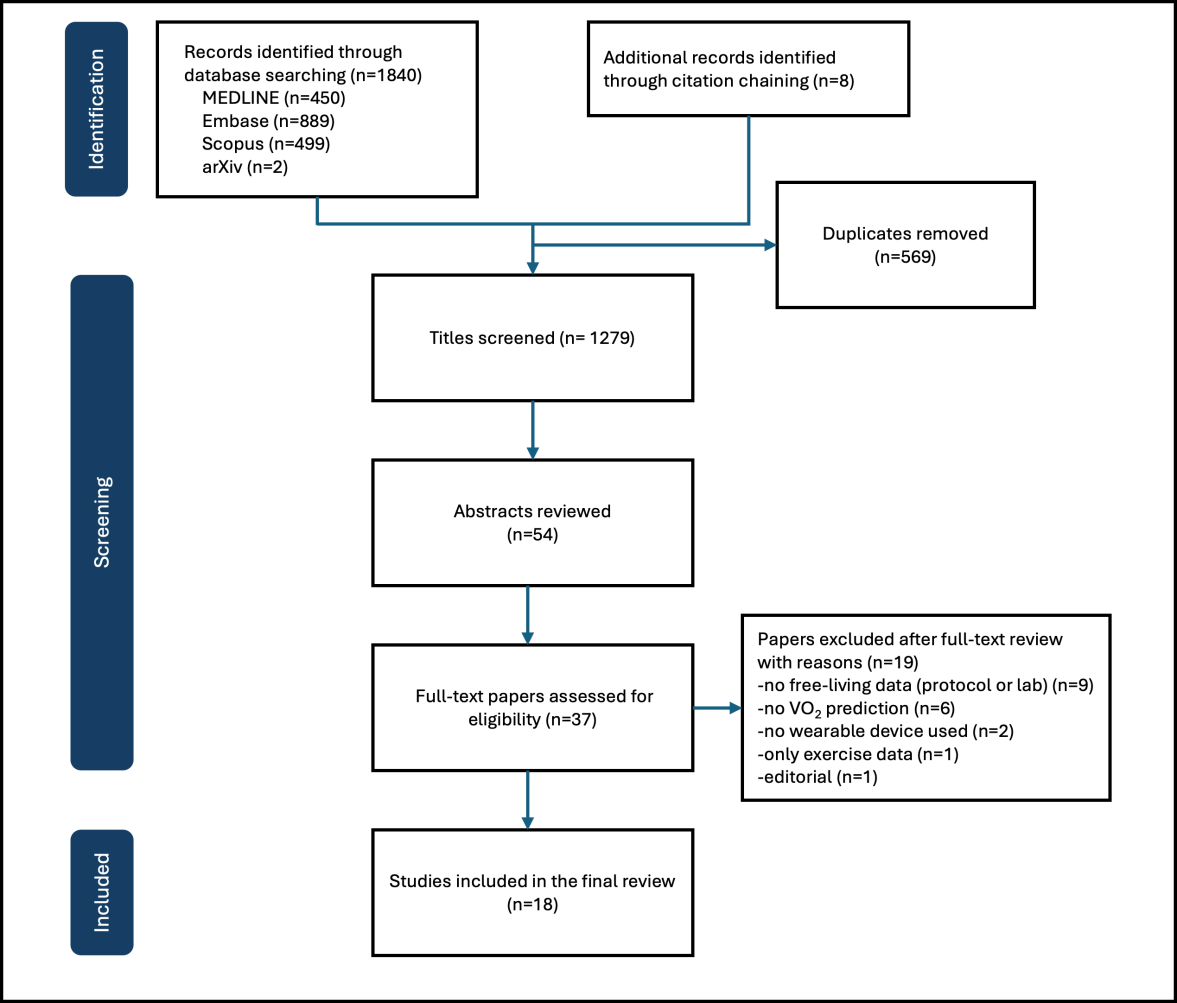
Flowchart of study selection following the PRISMA (Preferred Reporting Items for Systematic Reviews and Meta-Analyses) guidance. VO_2_: maximal oxygen uptake.

The combined literature search generated 1848 papers, with 1279 remaining after deduplication. The PRISMA flowchart (Figure 1) shows the paper selection process. Following title and abstract screening, 37 papers qualified for full-text review. Eighteen studies were accepted in the final set with a total sample size of 31,072 participants. Sample sizes varied greatly across studies and ranged from 13 to 12,425. Only 4 studies had a sample size of more than 1000 patients, indicating that most research in this field is based on a relatively small number of participants [27-30].

The characteristics of each included study are shown in Table 1 [2,4,5,7,11,12,16,27-37]. Participant-level weighted mean age was 46.9 (SD 1.46) years, with a male participant distribution of 48.9%. Notably, a few studies had exclusively male or female participants, while others had more balanced samples. Most studies included data from volunteers recruited in a prospective manner (n=13, 72%). Smaller studies focused primarily on healthy participants (n=13), in contrast to the larger cohorts (n=5) that used data from population studies involving hundreds of patients (the Fenland and Framingham studies) [38,39]. Overall, there were only 2 research trials that targeted patients scheduled for preoperative assessment [4,7].

**Table 1.** Summary of study characteristics.

Authors	Year	Sample size (M^a^), n (%)	Age (years), mean (range)	Sensors and modality used	Wearable monitoring (days)	Participants	Control	Reference VO_2_, mean (SD)
Plasqui and Westerterp [2]	2005	25 (40)	28 (18-50)	Tracmor elastic belt triaxial accelerometer and Polar (S610i) HR^b^ monitor wristwatch	7 (daytime only)	Healthy volunteers	Maximal GXT^c^ cycle	M=49.5±10.2; F^d^=40.7±8.4
Plasqui and Westerterp [33]	2006	26 (53.8)	29 (18-50)	Tracmor elastic belt triaxial accelerometer and Polar (S610i) HR monitor wristwatch	7 (daytime only)	Healthy volunteers	Maximal GXT cycle	44.6±10.5
Cao [34]	2009	189 (0)	49.6 (20-69)	Kenz Lifecorder uniaxial accelerometer	7 (daytime only)	Healthy volunteers	Maximal GXT cycle	31.4±7.4
Cao et al [35]	2010	148 (0)	47 (20-69)	Kenz Lifecorder uniaxial accelerometer and triaxial accelerometer	7 (daytime only)	Healthy volunteers	Maximal GXT cycle	30.8±5.9
Novoa et al [4]	2011	38 (79)	62.8 (38-80)	OMROM Walking Style ProW accelerometer (two modes- aerobic mode after 10 min of walking at 60 steps per min)	7-41 (daytime only)	Patients scheduled for lung resection	Maximal GXT cycle and Arterial gas	20.3±4.6
Altini et al [11]	2016	46 (45)	24.7 (NR^e^)	ECG^h^ Necklace (one lead ECG) and ADXL330 triaxial accelerometer and Mobile phone for GPS coordinates	14 (during laboratory protocols and free-living)	NR	Maximal GXT cycle	44±9.8
Altini et al [5]	2016	51 (47)	NR^e^	ECG Necklace (one lead ECG) and ADXL330 triaxial accelerometer	14 (during laboratory protocols and free-living)	Healthy volunteers	Maximal GXT cycle	NR^e^
Beltrame et al [37]	2017	13 (100)	26 (NR)	Hexoskin smartshirt (hip accelerometer, three lead ECG and respiration bands)	4 (free-living 9 AM to 5 PM) and simulated ADLs^f^	Healthy volunteers	Simulated ADLs and Pseudorandom Ternary sequence.	NR
Ahn et al [31]	2017	24 (100)	27.5 (NR)	Shimmer ECG sensor (18), for measurement of 2-lead ECG and tri-axial accelerometer	4 (daytime only)	Healthy volunteers	Maximal GXT treadmill	48.5±5.3
Kwon et al [12]	2019	240 (47)	42 (20-65)	Fitbit (Fitbit Charge; Fitbit) (triaxial accelerometer, PPG)^g^	3 (daytime only)	Healthy volunteers	Maximal GXT treadmill	36.25
Bonomi et al [16]	2020	40 (48)	25 (18-55)	Chest-belt HR ECG monitor (RS800CX, Polar, Wrist activity monitor (Tracmor)	5 (daytime) and simulated ADLs	Healthy volunteers	Maximal GXT cycle	M=45.7±6.1; F=40±6.6
Jones et al [7]	2021	49 (65)	65 (NR)	Garmin Vivosmart HR+ activity tracker wristwatch (PPG, accelerometer, GPS)	7 continuous	Patients for preoperative assessment	Maximal GXT cycle	18.2±4.5
Spathis et al [30]	2021	2100 (46)	48.7 (35-65)	Combined HR and uniaxial movement sensor (Actiheart) and wrist triaxial accelerometer	6 continuous	Population-based cohort study (Fenland)	Submaximal GXT treadmill	NR
Wu et al [28]	2022	12,425 (47) 181	47.7 (35-65)	Combined HR and uniaxial movement sensor (Actiheart) and wrist triaxial accelerometer	6 continuous	Fenland study population-based cohort and UK Biobank	Submaximal GXT treadmill Maximal GXT test	NR; 32.95
Spathis et al [27]	2022	11,059 (47)	47.7 (35-65)	Combined HR and uniaxial movement sensor (Actiheart) and wrist triaxial accelerometer	6 continuous	Fenland study population-based cohort	Submaximal GXT treadmill	M=41.95±4.61; F=37.44±4.73
Frade et al [32]	2022	43 (74.4)	37.5 (19-72)	Hexoskin smartshirt (hip accelerometer, three lead ECG and respiration bands)	7 (daytime only)	Volunteers (chronic disease allowed)	Maximal GXT cycle	32.09
Neshitov et al [29]	2023	3894 (67)	42 (20-65)	Apple Watch and Garmin watch (PPG, triaxial accelerometer, GPS)	mean 287, SD 149	Healthy volunteers -consented to Welltory app	Estimated by smartwatch device	36.16±6.66
Zhang et al [36]	2024	662 (41)	53 (NR)	Apple Watch (PPG, triaxial accelerometer, GPS)	mean 128 (daytime only)	Framingham study cohort	Maximal GXT cycle	M 27±7; F 22±6

a M: male.

b HR: heart rate.

c GXT: graded exercise test.

d F: female.

e NR: not reported.

f ADL: activities of daily living.

g PPG: photoplethysmogram.

h ECG: electrocardiogram.

A wide variety of wearable devices were used, including triaxial accelerometers, ECG sensors, and smartwatches with optical photoplethysmogram sensors. All studies used accelerometers for motion tracking, while HR monitoring was performed either with ECG (n=9) or optical sensors (n=6). The monitoring durations also differed, with most studies tracking participants over a few days, usually 3‐7, while others extended up to nearly a year [29]. Measurement of VO_2_max as the ground truth was typically obtained through a maximal CPET test, though in 4 trials, a submaximal treadmill test was used. In 1 study, reference VO_2_max was not directly measured but estimated using the proprietary algorithm of a smartwatch device [29]. Due to disparities in participant populations, the reference mean VO_2_max varied significantly between patient-centered studies (weighted mean VO_2_max 19.2 mL/kg/min, SD 1.48) and healthy volunteer studies (40.2 mL/kg/min, SD 6.58; z test, *P*=.03).

### Features

Feature extraction is a crucial first step in signal processing, transforming complex raw data points into meaningful numerical features that can be interpreted and processed in a model [40]. In high-volume continuous wearable data, feature extraction can also help to reduce dimensionality without losing important information. This process makes data handling easier and speeds computation by focusing only on the most relevant aspects of the data [41]. We observed, however, that in 6 studies, researchers adopted features as reported from the internal proprietary algorithm of the manufacturer without further analysis of the raw data. Table 2 summarizes the analytical methods used in the included studies, while Table 3 provides an overview of each feature type with examples in the studies used.

**Table 2.** Summary of analytical methods, models, and wearable features used in the included studies.

Study, year	Wearable features	Model used	Preprocessing techniques and analysis	Model performance	Validation	Principal finding
Plasqui and Westerterp [2], 2005	^a^HR/ACM^b^ index (ACM: activity counts per minute)	Multiple linear regression	Minute averages for HR and activity counts averaged over the 7 days, ratio of HR/ACM, and missing data removed	SEE^c^=12.4%; *r*=0.87	NR^d^	Fitness index HR/ACM significantly related to VO_2_max^e^ corrected for body composition and age
Plasqui and Westerterp [33], 2006	HR/ACM index	Multiple linear regression 2nd equation tested	Same as above and groups combined and sorted for activity counts	*r*=0.86; SEE=10.7%; Bland-Altman systematic error=5.6%	Cross-validation	A second equation for the fitness index HR/ACM had to be tested to predict VO_2_^f^
Cao et al [34], 2009	Daily SC^g^	Hierarchical linear regression	Steps per day provided and handling of missing data not reported	SEE=10.9%; *r*=0.81	Split test	SC was a significant contributor to the prediction of the measured VO_2_max
Cao et al [35], 2010	MVPA^h^, VPA^i^, and SC	Hierarchical linear regression	As provided (minutes spent in MVPA and VPA) and handling of missing data not reported.	SEE=9.66%; *r*=0.863	Cross-validation, Subgroup analysis	VPA significantly increased the explained variance in VO_2_max, adjusted for age
Novoa et al [4], 2011	Daily SC, Aerobic SC, time spent in aerobic activity, and daily distance in km	Linear regression	As provided and handling of missing data not reported	*R*^2^=0.93	Bootstrapping (1000 iterations)	VO_2_max can be signiﬁcantly predicted by the mean daily walked distance
Altini et al [11], 2016	HR at different walking speeds, stay regions, and activity composites (ie, HR at relative time spent in each activity)	Nonnested hierarchical Bayesian regression, SVM^j^ classiﬁer, and HMM^k^ for transitions between activities	LDA^l^ activity is classified into 6 clusters, accelerometer data was band-passed between 0.1 and 10 Hz to isolate dynamic components, HR was extracted from R-R intervals and averaged over 15 seconds, and missing data not analyzed	*R*^2^=0.76; RMSE^m^=249.4; SEE=5.79%	Leave-one-participant-out cross-validation	Contextualizing HR by means of activity and speed improved correlation between free-living HR and CRF^n^
Altini et al [5], 1985	HR/min while lying down and while walking at 3.5 and 5.5 km/h	Multiple Linear regression, SVM classiﬁer	HR was extracted from R-R intervals and averaged over 15 seconds windows and unusable ECG^o^ was discarded. Acceleration signal was segmented in nonoverlapping intervals of 5s	*R*^2^=0.78; RMSE=284.7	Leave-one-participant-out cross-validation	Submaximal context-speciﬁc HR can be used to estimate VO_2_max
Beltrame et al [37], 2018	Means of HR, VE^p^, BF^q^, hip acceleration, and SC in the 2 conditions (“active-inactive”)	Random forest	HR was averaged every 16 beats, VE and BF average of the last 7 respiration cycles, all features were time-aligned, low-pass ﬁltered at 0.01 Hz. Fast Fourier transformation and frequency domain analysis for hip acceleration, and when hip acceleration was >0.05 g, data were labeled as “active”; otherwise, they were “inactive	*r*=0.88	Leave-one-participant-out cross-validation	Predicted oxygen uptake data during ADLs^r^ were strongly correlated with the temporal characteristics of the VO_2_ during a controlled protocol
Ahn et al [31], 2017	aEE^s^ (nonlinear model derived from ACM horizontal and ACM vertical signals and HR per minute	Linear regression between HR and aEE	The tri-axial acceleration was band-pass filtered (0.25 to 7 Hz). ECG data were band-pass filtered (5 to 20 Hz), the R-R intervals were averaged for 1 min and converted to a HR, and used only increasing HR periods and excluded data with inaccurate ECG	*R*^2^=0.74; *r*=0.87; SEE=11.85%	Split test	aEE can be used to estimate VO_2_max during daily activities
Kwon et al [12], 2019	HR and daily PA in terms of METs, used slope of HR and PA	Linear regression	Moving average ﬁlter was applied, data points at which both HR and physical activity data increased were selected	*R*^2^=0.651; SEE=3.518; 9.6%	PRESS^t^ for cross-validation	VO_2_max can be estimated using novel features, aEE and the slope between physical activity and HR
Bonomi et al [16], 2020	Acceleration and HR- fitness index named TEE-pulse^u^	Stepwise linear regression	Motion intensity was deﬁned as activity counts per minute, acceleration signal was processed in overlapping windows of 60 seconds, and activity is grouped in (sedentary, or other) based on a set of counts thresholds	RMSE=367 or 12.4%; SEE=13.09%; *r*=0.89; MAE=10.2%	Leave-one-participant-out cross-validation	The daily average TEE-pulse was highly correlated to the mean TEE-pulse measured in the laboratory without the need for specific exercise protocol
Jones et al [7], 2021	Floors climbed, total number of steps and total distance, average HR and resting HR	Linear regression	Features were used as provided by the device and averaged across the 7-day wear period. Self-reported METs^v^ from questionnaires	AIC^w^=181.62; *R*^2^=0.74; *r*=0.86; AUC^x^=0.93	NR	Using all the wearable variables together in linear regression gave a stronger correlation between the measured CPET^y^ values, specific- ally for peak VO_2_
Spathis et al [30], 2021	HR per minute, acceleration (magnitude calculated through ENMO^z^), resting HR	Step2Heart Deep neural network, (CNN^aa^ learn spatial and RNN^ab^ temporal features)	Noisy heart data removed with a Gaussian process robust regression, participants with less than 72 hours of wear were removed, and accelerometry and ECG signals were summarized to a common time resolution of one observation per 15 seconds	AUC=0.70; RMSE=9.54 (HR forecasting only)	Split test	A general-purpose self-supervised feature extractor for wearable data was developed. HR forecasting transfer learning of learned physiological representations
Wu et al [28], 2022	HR and movement 26 features combining HR, movement data, and time-series metadata	UDAMA^ac^	Nonwear periods were removed (periods of nonphysical HR and no movement), downsampled the sampling rate to 15 minutes and used the ﬁrst 600 timesteps, and pretrained on noisy data and used adversarial training on the BBVS dataset	*R*^2^=0.392; *r*=0.665; MSE^ad^=30.79; MAE^ae^=4.44	Split test and 3-fold cross validation	A novel model proposal to leverage noisy data from source domain (wearable dataset) to improve modeling for accurate fitness estimation at scale
Spathis et al [27], 2022	48 features: Raw acceleration derived through ENMO, HR, HRV^af^, MVPA for each feature mean, minimum, maximum, SD, and the slope of a linear regression ﬁt	Deep neural network- adaptive representation learning	Nonwear periods removed, movement intensities were converted into standard METs, principal component analysis for noise reduction, tSNE^ag^, a nonlinear dimension-reduction technique was applied	*R*^2^=0.658; RMSE=2.956; *r*=0.82; RMSE=8.998 (Biobank only)	Split test and External validation in UK biobank cohort (181 patients)- Maximal GXT^ah^ testing	A deep learning frame- work for predicting CRF was developed, combining learned features from HR and accelerometer free-living data without context awareness
Frade et al [32], 2023	Mean HR and BF, minute ventilation, tidal volume, mean hip acceleration, and mean SC	SVM (support vector regression formulation)	Abnormal HR and BR^ai^ were excluded with a preprocessing algorithm (not mentioned), and all raw data were averaged	*r*=0.804; MAE=3.84	k-fold cross-validation	Hemodynamic domain presented statistically higher importance to predict the VO_2_max compared with activity and Pulmonary domains
Neshitov et al [29], 2023	HR and SC/min, HR/cadence ratio, daily MET, and HR response to cadence increase	Quantile regression (for each quantile a gradient boosting model was trained)	The HR stream was resampled to 1 measurement per minute and averaged over consecutive 1-minute intervals, cadence is the number of steps made during the same 1-minute interval, continuous ranked probability score used for hyperparameter tuning, and model trained on estimated VO_2_ from wearable device	Test set: ECE^aj^=0.032; IQR=3.948; MedPE^ak^=0.01; and Direct VO_2_ dataset: ECE=0.084; IQR=4.705; MedPE=0.35	Split test for training External validation (10 healthy volunteers) Maximal GXT treadmill	Anthropometric characteristics were the most influential feature, followed by cadence to HR ratio. The proposed model provides a point estimation and a probabilistic prediction of VO_2_; to estimate the prediction’s uncertainty
Zhang et al [36], 2024	Daily SC, mean HR	Multivariable linear regression, sensitivity analysis for age, BMI, gender	Defined HR as nonactive if recording interval was >1 minute, hourly steps <30, inferred motion context status for HR measures that lacked motion, and excluded days with <5 hours of wearing time	*R*^2^=0.07‐0.12	NR	Every 1.3 mL/kg/min higher peak VO_2_ corresponded to a 2.4-bpm lower nonactive HR. Physical activity with 1.3 mL/kg/min higher peak VO_2_ was associated with nearly 1000 more daily steps

a HR: heart rate.

b ACM: activity counts per minute.

c SEE: standard error of estimate.

d NR: not reported.

e VO_2_max: maximal oxygen uptake.

f VO_2_: measured oxygen uptake.

g SC: step count.

h MVPA: moderate to vigorous physical activity.

i VPA: vigorous physical activity.

j SVM: support vector machine.

k HMM: hidden Markov model.

l LDA: latent Dirichlet allocation.

m RMSE: root-mean-square error.

n CRF: cardiorespiratory fitness.

o ECG: electrocardiogram.

p VE: minute ventilation.

q BF: breathing frequency.

r ADL: activities of daily living.

s aEE: activity energy expenditure.

t PRESS: predicted residual error sum of squares.

u TEE: total energy expenditure.

v MET: metabolic equivalent task.

w AIC: Akaike Information Criteria.

x AUC: area under the receiver operating characteristic curve.

y CPET: cardiopulmonary exercise testing.

z ENMO: Euclidean norm minus one.

aa CNN: convolutional neural network.

ab RNN: recurrent neural network.

ac UDAMA: unsupervised domain adaptation via multidiscriminator adversarial training framework.

ad MSE: mean squared error.

ae MAE: mean absolute error.

af HRV: heart rate variability.

ag tSNE: t-distributed stochastic neighbor embedding.

ah GXT: graded exercise test.

ai BR: breathing rate.

aj ECE: expected calibration error.

ak MedPE: median prediction error.

**Table 3.** Wearable features grouped by type, with examples used in each of the included studies.

Feature type	Studies	Examples
Motion	Cao et al [34] and Cao et al [35] Novoa et al [4] Beltrame et al [37] Jones et al [7] Spathis et al [27] and Spathis et al [30] Frade et al [32] Zhang et al [36]	Daily step count Moderate-to-vigorous physical activity Vigorous physical activity daily distance, mean hip acceleration, and acceleration magnitude
Cardiac	Altini et al [11] Beltrame et al [37] Ahn et al [31] Jones et al [7] Spathis et al [30] Frade et al [32] Zhang et al [36]	Average HR^a^ and resting HR HR/min Heart rate variability measures
Contextualized HR	Plasqui and Westerterp [2] and Plasqui and Westerterp [33] Altini et al [5] and Altini et al [11] Bonomi et al [16] Kwon et al [12] Wu et al [28] Spathis et al [27]	HR/ACM^b^ ratio index HR response to cadence increase activity composites (HR at relative time spent in an activity) HR at different walking speeds slope of HR and physical activity HR/cadence ratio
Other	Beltrame et al [37] Ahn et al [31] Kwon et al [12] Frade et al [32] Neshitov et al [29]	V_E_^c^, BF^d^, and tidal volume aEE^e^ TEE^f^ MET^g^

a HR: heart rate.

b ACM: activity counts per minute.

c VE: minute ventilation.

d BF: breathing frequency.

e aEE: activity energy expenditure.

f TEE: total energy expenditure.

g MET: metabolic equivalent.

### Motion Features

Activity features from the accelerometer data included mainly daily step count (SC), distance covered, time spent in anaerobic or sedentary activity (stay regions), and acceleration or walking speed (Table 3). From these, the most frequently reported feature was the daily SC, which in most instances was precalculated from the accelerometer’s proprietary algorithm. We found that some researchers reported steps as an average across several days, removing the temporal context which might be useful in analyzing trends [2,4]. Intensity of movement and walking speed was described as time spent in sedentary or vigorous activity, which can be calculated from the acceleration as activity counts per minute [16]. One study used simulated daily activities to correlate aerobic dynamics of variable intensity [37]. Finally, distance covered was either extracted directly from the device or computed as the total number of steps taken in a day multiplied by the stride length of the participant [4].

### Cardiac Features

Features extracted from the ECG and optical sensors included average HR, resting HR, HR per minute, mean HR, and ΔHR (difference between current and previous HR value to capture the magnitude of changes in cardiac activity). ECG signals are generally complex; they are subject to motion artifact, creating noise that affects quality [42]. Various filtering methods were applied to reduce noise. Researchers commonly use a band-pass filter between 5 and 10 Hz to remove artifacts and enhance the detection of the heartbeat from the R-R intervals. The R-R intervals were usually averaged over set-time windows, discarding any inaccurate values [11,31]. Beltrame et al [37] averaged HR every 16 beats, passing HR data through a low-pass filter at 0.01 Hz, removing high frequencies.

### Contextualized HR

The combination of HR and activity data, often referred to as contextualized HR, was reported in more recent studies. This contextual dimension of wearable signals can be vital in understanding the physiological cardiac response to exercise. Such features comprised HR at variable-intensity walking speeds, HR and SC per minute, HR/cadence ratio, and HR response to cadence increase (Table 2) [5,27,29]. Kwon et al [12] examined the slope between the concurrent increase in physical activity and HR, while others studied time-series metadata of HR and movement signals, mining numerous features [27,28]. Advanced signal processing methods, such as principal component analysis and fast Fourier transform, were implemented to tackle noisy heart data, but this was not standardized across the included studies. Authors frequently used resampling techniques (standardizing time intervals between data points) to align HR with movement data, helping to contextualize HR within the corresponding physical activity [30].

### Other Features

Less common features included energy expenditure estimates in terms of metabolic equivalents, which were calculated in 3 studies based on daily physical activity and proprietary algorithms [12,16,29]. On 1 occasion, breathing frequency and minute ventilation were extracted as the average of the last 7 respiration cycles, based on respiration bands integrated into the wearable device used for monitoring [37].

### Models

Multiple modeling techniques were used among the included studies, with more advanced ML models such as support vector machines (SVMs) and deep learning gaining interest over regression in recent studies. This trend follows the usage of preprocessing techniques such as fast Fourier transform and frequency domain analysis and represents an effort to mine the raw data and uncover hidden patterns. A detailed breakdown of modeling and preprocessing techniques is provided in Table 2.

Eleven studies used linear models, which allow interpretation of outcomes through reporting of coefficients that give direct insights into how each predictor influences the outcome measured [2,4,7,11,12,16,31,33-36]. The earliest study that examined whether the ratio of HR to activity counts could predict VO_2_max was from Plasqui and Westerterp [2]. Two studies used correlation analysis to identify the strongest predictors of VO_2_max and built on this using linear regression [4,7]. The rest of the studies outlined combinations of modern ML techniques. Three studies reported SVMs, with Frade et al [32] presenting support vector regression, which is an SVM formulation for regression problems. SVMs are supervised models that identify an optimal decision boundary (hyperplane) to separate data points into distinct classes with the aim of maximizing the margin between observed and predicted values.

Beltrame et al [37] were the only group to consider a random forest model. Random forests aggregate several decision trees together as a group but introduce randomness to prevent overfitting [37]. Another study trained several gradient boosting models and then fitted a quantile regression algorithm to predict the distribution of VO_2_max with CIs [29]. In boosting, models are trained sequentially, building on the errors or residuals of the previous model to improve their prediction accuracy.

Finally, 3 studies [27,28,30] leveraged large-scale free-living datasets to predict CRF with variations of neural networks and deep learning. Wu et al [28] introduced a novel 2-stage approach building an adversarial training framework based on unsupervised domain adaptation. The proposed model was pretrained with noisy health-related labels in a fully supervised setting to improve its performance on high-quality, gold-standard data. Coarse- and ﬁne-grained discriminators were used to better handle the distribution shifts between source (silver-standard) and target (gold-standard) datasets. Spathis et al [27] applied principal component analysis to denoise the raw data and developed deep neural network models able to capture nonlinear relationships between numerous wearable features and VO_₂_max.

### Length of Available Data Required for Prediction

Some studies examined the minimum length of free-living wearable data that would be required to reach reliable conclusions regarding VO_2_max estimations, but no agreement was observed. The most thorough assessment was provided by Neshitov et al [29], who tested the degree of certainty for 5 different models using various amounts of available data. They advocated that a minimum of 200 minutes is required for an error estimation range of 4.5 mL/kg/min, but more than 1000 minutes is needed to improve this to under 4 mL/kg/min. Ahn et al [31] plotted the correlation coefficient values with the included measurement time and found no drastic improvements between VO_₂_max and estimated values past the 900-minute mark, which yielded an *r* value of 0.81. On the contrary, other researchers reported that even 10 minutes per day of good-quality data might be sufficient to predict VO_2_max. Beltrame et al [37] determined the 10-minute window from the frequency domain analysis as the ideal size for data extraction based on iterative testing of different window lengths (200-1000 seconds, incrementing by 100 seconds). A window length of 600 seconds (10 minutes) was found to provide the best balance between maximizing frequency resolution and ensuring enough reliable samples for analysis across participants. Altini [5,11] proposed this on a theoretical basis, as many submaximal protocols are of a 10-minute duration (eg, 6-minute walking test). Other studies did not account for a minimum length but excluded patients with <72 hours of data from the analysis [27]. Ultimately, this large disparity in the length of data required for feature engineering reflects the exploratory nature of some of the included studies.

### Quality of Studies

**Figure 2.**
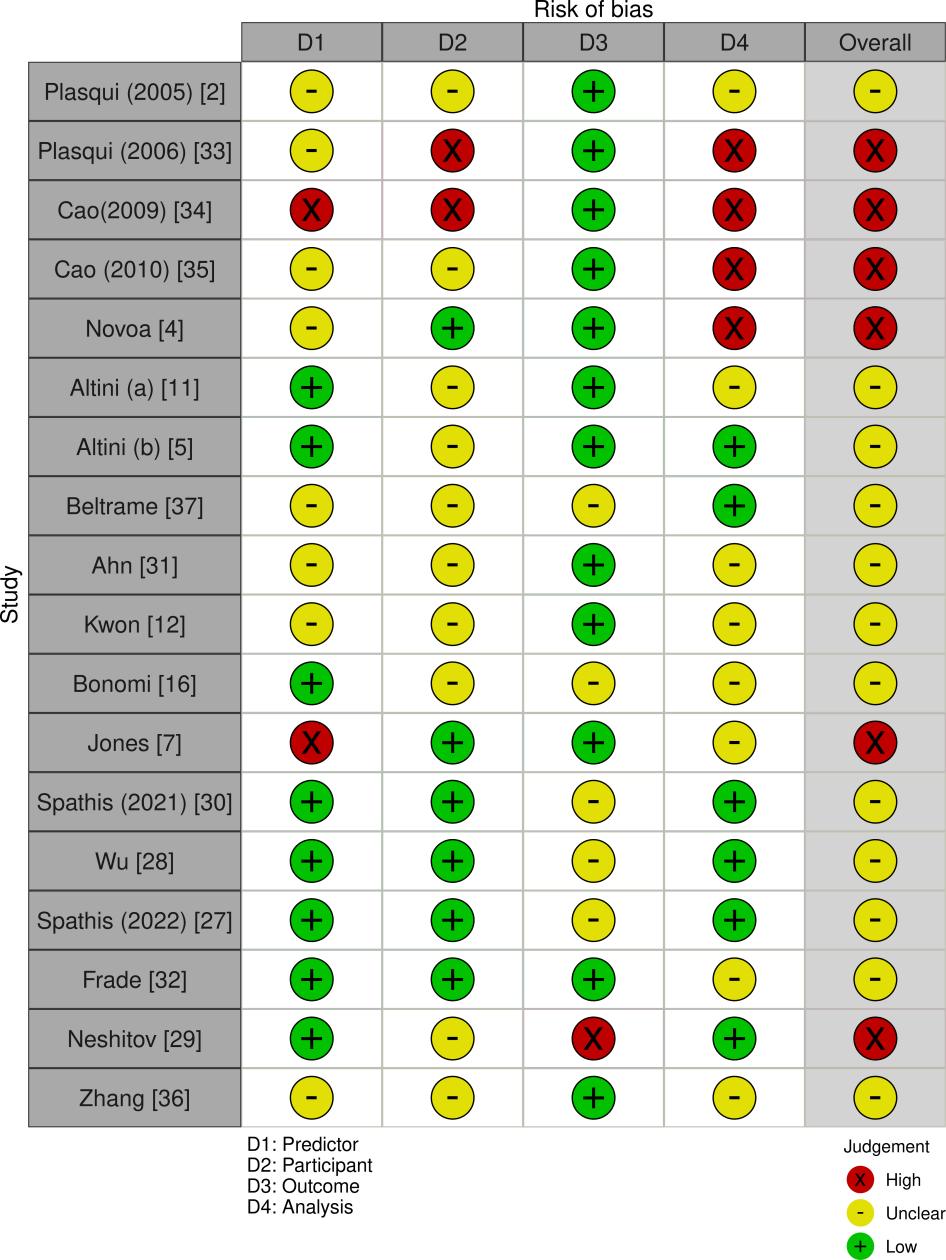
Risk of bias distribution among the included studies [2,4,5,7,11,12,16,27-37].

Risk of bias distribution for each domain is provided in Figure 2 [2,4,5,7,11,12,16,27-37]. Critical appraisal of the included papers showed that only 1 study was classified as “low risk of bias” for all domains. CRF was aptly measured as the outcome of interest in 12 (67%) studies using data from maximal exercise tests (also provided in Multimedia Appendix 2). Appropriate selection of participants and predictors was documented in 6 and 8 studies, respectively. Higher degrees of bias were observed in the analysis domain with robust reporting of analytical methods noted only in 6 (33%) studies. Handling of missing data was not reported adequately in 5 studies [4,5,11,34,35], while others excluded data from analysis arbitrarily, without fully justifying their decisions [12].

Most studies (n=13) reported internal validation methods for their predictive model, such as split-test or leave-one-participant-out cross-validation (Table 2). Model validation was not considered in 3 studies, and only 2 papers tested their algorithm externally on unseen data [27,28].

### Model Performance

**Figure 3.**
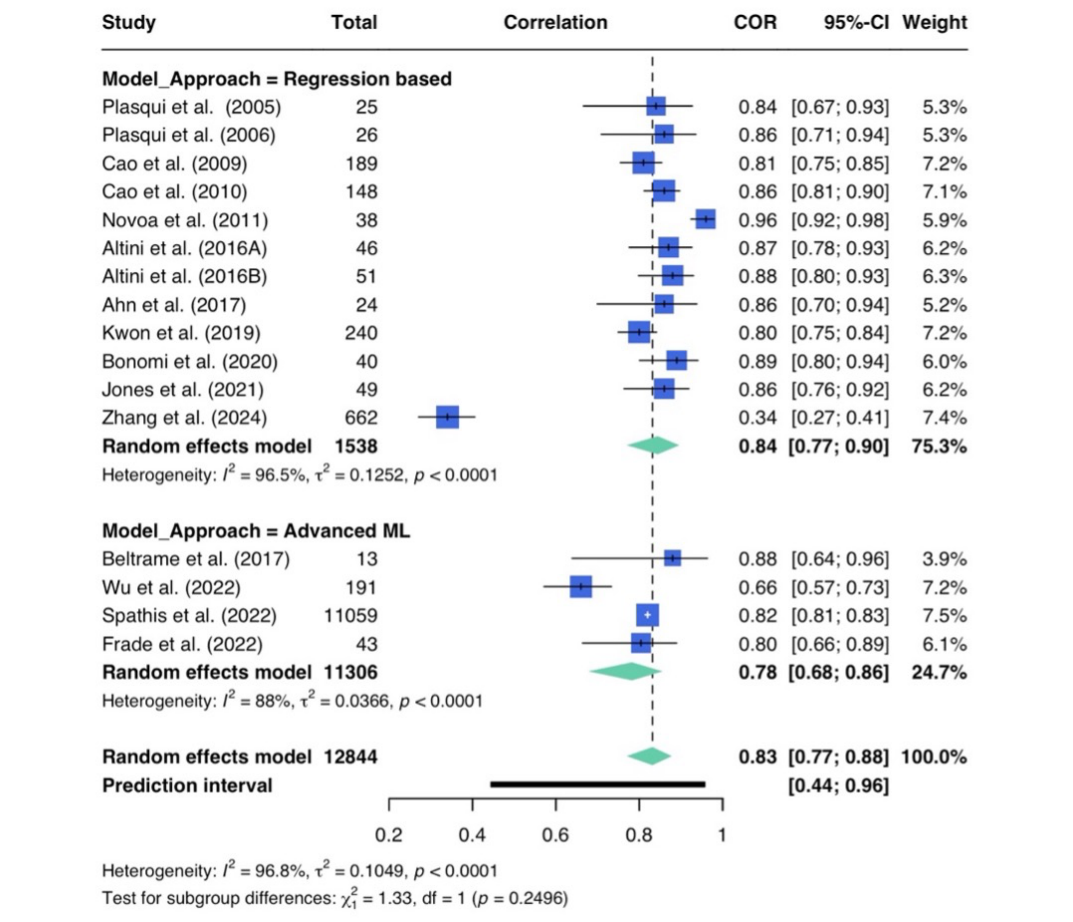
Forest plot of the meta-correlation analysis between maximal oxygen uptake estimates and reference values. Random-effects study weighting was calculated as the inverse sum of the in-study variance and the between-study variance (τ^2^). Subgroup analysis comparing modeling approaches is also presented [2,4,5,7,11,12,16,27,28,31-37].

Various model performance metrics were reported (Table 2). The weighted average SEE was 9.03%, indicating that models overall predict VO_2_max with an error of approximately 9%. In total*,* 16 studies were included in the meta-correlation analysis, which is provided in Figure 3 [2,4,5,7,11,12,16,27,28,31-37]. The pooled overall estimate of *r*=0.83 with a 95% CI of 0.77-0.88 from the random-effects model indicates a positive agreement between predicted and observed VO_₂_max values. Heterogeneity among the included studies was high, with *I*²=97% and a Q test of statistical significance (*P*<.01). Furthermore, moderate variance was observed (τ²=0.1049), suggesting underlying differences in how well VO_₂_max is predicted across studies. Subgroup analysis comparing regression-based methods with more advanced ML methodologies favored regression, but the difference was not statistically significant (*P*<.24; Figure 3 [2,4,5,7,11,12,16,27,28,31-37]).

**Figure 4.**
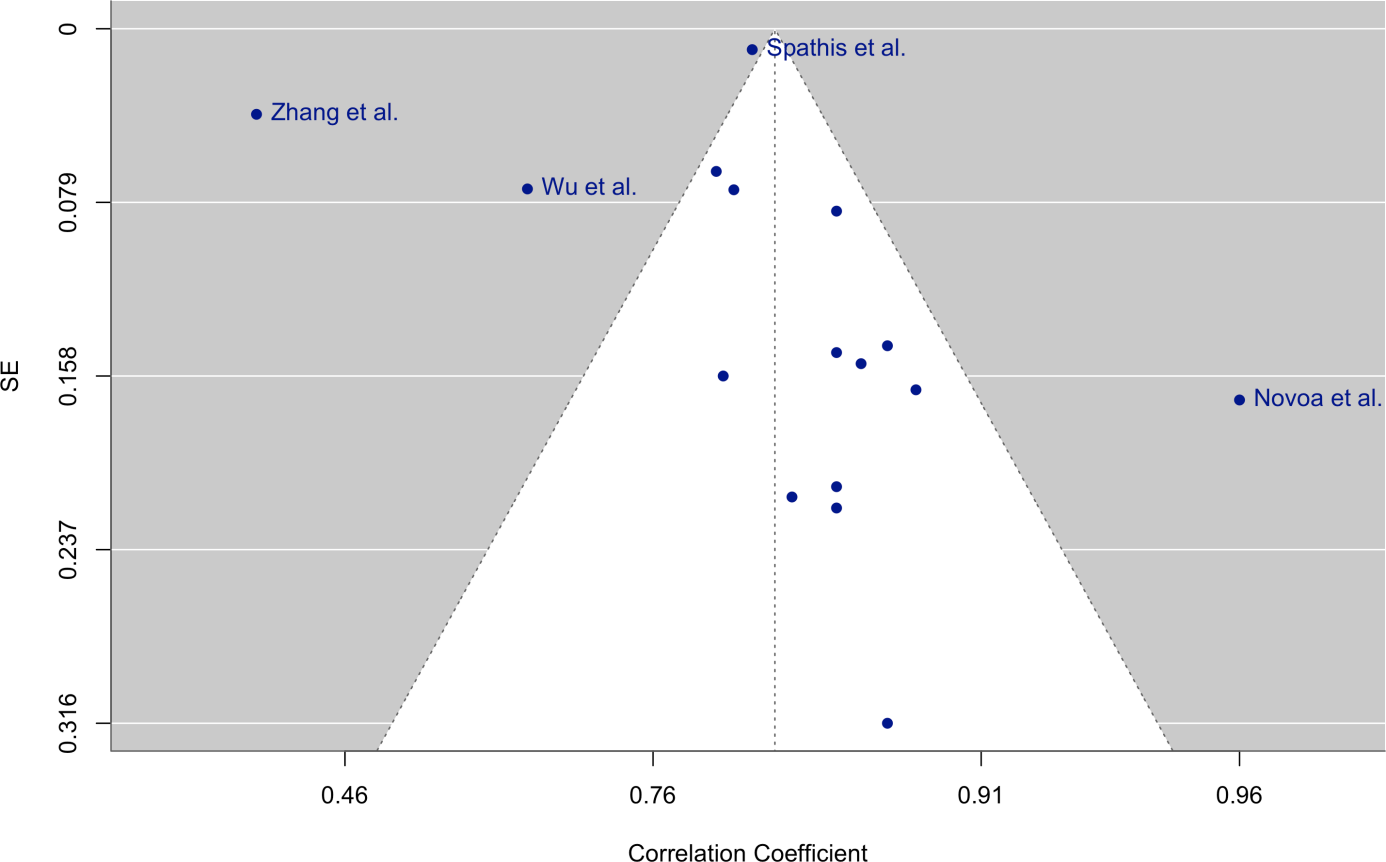
A funnel plot illustrating the distribution of study effect sizes to assess potential publication bias. Four studies fall outside the expected range, suggesting potential publication bias [4,27,28,36].

In studies reporting high correlations, there are several common features. While most research indicated the use of validation methods with unseen data (train-test split and cross-validation) to test model performance, several studies reported the *R*² values from the linear regression model [4,7]. Another common factor among the highest-performing models was the incorporation of data collected from laboratory protocols, either to contextualize or interpret free-living data, into feature extraction and modeling processes [5,11,16]. In addition, the funnel plot provided in Figure 4 [4,27,28,36] revealed asymmetry with 4 outliers, which was also confirmed with an Egger test (z=2.29; *P*=.02). This suggests the presence of small-study effects and publication bias. Notably, the overoptimistic, smaller-sized study by Novoa et al [4] is likely overrepresented in the pooled estimate, and results need to be interpreted with caution.

## Discussion

### Principal Findings

This systematic review identified research using real-world, unsupervised wearable data to develop predictive models for CRF estimation, focusing on VO_₂_max as the measure of interest. Our study adds to the literature as the first to appraise evidence in this field and showcase the ability of advanced ML algorithms to harness the power of unstructured physical activity outside controlled laboratory settings. The included meta-correlation analysis revealed a pooled overall estimate of 0.83 with a 95% CI of 0.77-0.88, and a mean SEE of 9.06%, demonstrating a promising overall agreement between predicted VO_2_max and ground truth. Authors experimented with a range of sensor modalities and various population groups, but models were predominantly designed based on small-sized, healthy volunteer data. Several features were extracted from the free-living information, including SC and distance covered, resting and mean HR, and cardiac response to cadence increase. Quality control of the eligible studies showed that authors were consistent in predictor and outcome reporting, but analytical methods were often ambiguous and included some arbitrary decisions regarding data manipulation.

### Advantages

The concept of CRF estimation based on free-living activity holds considerable potential, and results from this study suggest that this could be a pragmatic alternative to CPET. Leveraging longitudinal wearable data can aid preoperative risk assessment for frail patients or those with musculoskeletal conditions that underperform during CPET (indicated usually by a respiratory exchange ratio of <1.10) [8]. Researchers have argued that physiological signals captured over longer time periods may even be more representative of cardiac health in these populations [5,37] compared to a snapshot laboratory measurement. In addition, at-home monitoring offers a convenient and unintrusive assessment without the need for speciﬁc protocols, improving patient experience and reducing the psychological stress related to the hospital environment [43]. Considering decreasing costs and increasing accessibility [44], continuous monitoring could represent a complementary, more cost-effective, efficient method that can be scaled to accommodate all patients [7]. Serial measurements of VO_2_max can not only help patients track progress and meet targets set during prehabilitation and rehabilitation programs but also guide clinician decision-making [45].

CRF is a well-established marker of CVD and all-cause mortality [3]. Considering that low levels of CRF may precede the clinical detection of CVD, early recognition and intervention are of patient benefit [37]. Wearable-driven evaluation of the aerobic response during unsupervised activities of daily living holds prognostic value in tracking changes in fitness over time, as demonstrated by Spathis et al [27]. Arguably, models that predict future CRF levels could help identify early-stage CVD before general symptom manifestation [37]. Finally, scrutinizing free-living data with advanced ML presents a rare opportunity to study patient behavior and activity habits, shedding light on individual CRF levels [37]. But above all, tailored interventions can be implemented promptly to improve patient fitness and outcomes [46].

### Contextualizing HR

The advent of wearables is undoubtedly transforming the landscape of health monitoring, providing clinical teams with a substantial amount of user-generated time-series data [27]. Although some earlier published studies used aggregates over several days as features (average steps or HR data), potentially losing information on trends and variability [2,33-36], most researchers in this review worked on extracting features from the raw signals, with seven studies aligning cardiac and activity points to contextualize HR and gain insights into the participants’ physiological response to workload. Based on this principle, Plasqui and Westerterp [2] were the first to publish a fitness index as a ratio between acceleration and HR. Studies of more advanced modeling included other multimodal features such as HR at certain speeds or activities and the HR response to acceleration and recovery [5,29,37]. As physical activity encompasses both body movement and an associated cardiovascular response, leveraging these signals concurrently allows for a better evaluation of the temporal dynamics of CRF and enhances the understanding of the individual’s physiology [27,37,47]. It should be emphasized, though, that contextualizing HR in unlabelled data requires navigating many intricacies, as investigators need to account for external factors that can influence HR, such as emotional stress, illness, heat, or medications that can potentially lead to invalid results.

In addition, a key observation arising from studying the multimodal features is the inverse relationship between VO_2_max and HR at a given physical activity, which conforms to what is seen during the submaximal tests [5,29,33]. This observation reinforces the concept of estimating fitness from free-living activity, as even in the absence of controlled settings, behaviors approximating submaximal laboratory conditions will spontaneously occur [47]. Neshitov et al [29] demonstrated this inverse relationship, with the slope of the HR-over-cadence regression line being lower for participants with high than those with low VO_2_max, and it was mainly noticeable between the 60‐100 steps per minute exercise effort. Interestingly, Bonomi et al [16] highlighted the need for activity-speciﬁc prediction equations, showcasing models that combined energy expenditure and HR based on different activity types. Ultimately, tailoring predictive models to account for specific activity patterns and physiological responses enhances the accuracy of CRF predictions.

### Challenges

Despite their potential, free-living data present some intrinsic statistical and computational challenges [48]. Using wearables in out-of-hospital, free-living settings often results in lower-quality, noisy data that require heavy filtering and preprocessing to become usable. Vigorous human motion can disturb on-body sensors and easily corrupt cardiac and accelerometer signals [40]. Aside from noise, missing data can also prevent meaningful features from being extracted. As reported in the “Results” section, preprocessing techniques are an essential step in the data-mining process to ensure that only reliable data points contribute to predictions. Another challenge lies in the precise physical activity detection in unlabeled data. Owing to the diverse nature of daily living, activity patterns overlap, and assumptions are occasionally made on how certain patterns in the data correlate with physical activities [5,49]. Furthermore, the abundance of sedentary data often leads to a data imbalance bias when low-intensity activities are overrepresented, leading to inaccurate estimations [5,16]. Consequently, due to the novelty of the task and the challenges outlined, there is currently no consensus on a specific approach and model that is most suited for free-living data.

### Regression and ML Approaches

Interestingly, in the subgroup analysis, regression-based models appeared to perform slightly better than ML approaches. Much research examining the 2 approaches has repeatedly demonstrated comparable performance between regression-based and ML approaches, but this is not universal [38,39]. Nonetheless, this finding from our review warrants attention, as it likely stems from issues such as reporting bias and overfitting rather than genuine superiority. Results are influenced by the notable disparity in sample sizes, with 1 study driving the pooled estimate in ML modeling studies [27]. Finally, the limited external validation in ML suggests that the robustness and interpretability of simpler models can, in some cases, outweigh the complexity when appropriate validation has not been considered.

### Limitations

This systematic review showed that free-living data can be valuable for CRF prediction and may prove a useful alternative in a variety of clinical settings. However, some limitations merit attention. First, although we observed promising preliminary agreement between VO_2_max estimates and predictions, we need to acknowledge that, although we chose the correlation coefficient as the primary effect size for the meta-correlation analysis for its availability, it is not an accuracy metric and therefore does not imply that predictions are close in absolute terms. Further, as in certain instances, conversion of the *R*^2^ was applied, this may have artificially inflated perceived predictive ability and, as such, influenced the overall result. Error-based metrics such as root-mean-square error or SEE would better capture accuracy, which is particularly relevant in clinical settings, but these measures were not consistently reported.

Second, there was significant heterogeneity and variance among the included studies, which varied in trial design, sample size, wearable device used, and modeling approach. Understandably, this limits the generalizability of our results, and the pooled estimate needs to be interpreted cautiously. However, in contrast to the synthesis of randomized trials, heterogeneity is frequently noted in meta-analyses of predictive modeling studies, mainly due to the disparity of eligible study designs or models [50].

Some studies used resting HR as a feature in their models, limiting monitoring to daytime periods only, excluding nocturnal HR data [31,32,36,37]. Research, however, demonstrates that using nighttime data can yield closer estimations to true testing values when it comes to resting HR [51]. As such, using daytime-derived resting HR may have implications for model performance and potentially lead to erroneous results. Godkin et al [51] underline the lack of standardization and considerable shortcomings among the criteria and methods used to estimate resting HR. Since daytime HR can be affected by numerous behavioral, psychological, and environmental factors, we advocate for continuous monitoring of free-living data that captures both activity and rest phases for a more stable profile of HR distributions.

Considerations should also be made when interpreting the outputs of the presented models, as the papers reporting the highest correlations between predicted and actual measurements share several common features. Notably, several studies presented outputs from regression models without using unseen data for validation. Directly comparing these outputs against models validated on unseen data likely overestimates the model’s true predictive ability. In such cases, high performance may reflect only how well the data fit the model, not its ability to generalize. Therefore, the lack of external validation may contribute to inflation in the aggregated meta-correlation analysis. Under such conditions, a single pooled correlation does not necessarily reflect a uniform level of accuracy and should certainly not be viewed as evidence of clinical readiness. Instead, it demonstrates the potential for future research that a strong association may be achieved.

Another limitation concerning the applicability and validation of the reported models is the selection bias, as most trials recruited young, healthy volunteers, making models less applicable to patient populations. We found that models predict VO_2_max with an average 9%, which, arguably, may be clinically relevant in borderline cases—for instance, during preoperative risk assessment of frail individuals. Consequently, no direct conclusions can be drawn for clinical decision-making, and future research should focus more on medical settings to assess the effect of patient-specific factors, such as regular medication and comorbidities, in model training. Interestingly, although there were several different devices used in the included trials, no study explored the practicalities of monitoring patients remotely to collect the free-living data, which could explain the quality issues of noise and missingness that these datasets exhibit.

### Implications for Real-World Use

The absence of a shared methodological framework across studies remains a major barrier to translation. As stated previously in the “Limitations” subheading, heterogeneity in this review was high, with studies using distinct device types, signal processing strategies, and validation tactics, often with insufficient external testing. Consequently, this limits confidence in generalizability and reproducibility, making direct comparisons difficult. From a clinical perspective, a model optimized for one setting may fail to transfer effectively in different patient groups, sensors, or wear patterns [52].

For clinical adoption to become feasible, several credibility issues need to be addressed. Pitfalls such as model overfitting, lack of standardized analytical pipelines, and limited evidence that performance is stable under real-world conditions (device updates, medication effects on HR) present significant challenges. Similar challenges have been identified in studies of prediction modeling for CVDs, highlighting the need for independent tools to assess replicability and external validation [52]. Therefore, until uniform data handling and transparent external validation become routine, results from research in this field should be considered promising but not ready for clinical application.

### Future Considerations

Despite the challenges and limitations identified in this review, several models reported here should not be overlooked in this expanding research field. Future research should aim to streamline the deployment of wearable devices in out-of-hospital settings and educate and support patients and clinical teams. Furthermore, given the increasing influence of the wearable industry in health care, it is essential for such predictive models to undergo rigorous validation before being fully integrated into clinical practice. Establishing consensus on feature extraction, validation, and reporting guided by frameworks such as TRIPOD-AI and recent calls for transparency in wearable research (INTERLIVE) are recommended for future research to yield reproducible and clinically useful results [21,53].

For public health systems, regulatory frameworks regarding the digital storage, privacy, and security of the vast amount of patient-generated data should be considered early. With the myriad of wearables available, it is important that feasibility work is undertaken to set standards for a reliable and accurate technology, helping avoid a repetitive cycle of temporary models being developed that cannot be extrapolated to clinical contexts or used in clinical practice. Finally, a cost-effective analysis will determine the viability of these remote monitoring systems, ensuring they offer a sustainable solution for patients and health care systems.

### Conclusion

This work explores a novel concept for CRF estimation from unsupervised free-living patient data. Contrary to the current gold-standard CPET, which is a snapshot of the individual’s functional capacity, wearable health monitoring in free-living conditions generates rich datasets that can be exploited to train models for fitness estimation. Several models are discussed in this paper, with studies applying ML to mine raw data and enhance accuracy.

The combined results from this review show promise, with good preliminary agreement between predictions and measured values. However, no firm conclusions can be drawn for clinical implementation due to the heterogeneity of the studies and the lack of external validation. Nonetheless, continuous data streams appear to be a valuable resource for ML methods to shed light on human behavior and health, leading to a step change in how we measure and monitor CRF, ultimately aiming to improve health outcomes.

## Supplementary material

10.2196/69996 Multimedia Appendix 1

10.2196/69996 Checklist 1 PRISMA (Preferred Reporting Items for Systematic Reviews and Meta-Analyses) checklist.

## Abbreviations

CPET: cardiopulmonary exercise testing
CRF: cardiorespiratory fitness
CVD: cardiovascular disease
ECG: electrocardiogram
HR: heart rate
ML: machine learning
PRISMA: Preferred Reporting Items for Systematic Reviews and Meta-Analyses
PROBAST: Prediction Model Study Risk of Bias Assessment Tool
SC: step count
SEE: standard error of estimate
SVM: support vector machine
TRIPOD-AI: Transparent Reporting of a multivariable or machine learning prediction model for Individual Prognosis Or Diagnosis–artificial intelligence
VO_₂_max: maximal oxygen uptake
