# Supplementary material for: Exploiting Unsupervised Free-Living Data for Cardiorespiratory Fitness Estimation: Systematic Review and Meta-Analysis
Source: JMIR Mhealth Uhealth. 2026 Jan 27;14:e69996. doi: 10.2196/69996 (PMC12841865; doi:10.2196/69996)
Supplement: Multimedia Appendix 1 [file mhealth-v14-e69996-s001.docx]

**Appendix S2**

**Scopus 27 July 2024**

( TITLE-ABS-KEY ( wearable OR ( fitness AND tracker ) OR accelerometer* OR pedometer OR smartwatch OR sensor OR biosensor )

1,794,157

AND

TITLE-ABS-KEY ( ( predict* ) OR ( estimat* ) OR ( calculat* ) )

13,827,506

AND

TITLE-ABS-KEY ( ( cardiorespiratory AND fitness ) OR ( vo2 OR vo2max OR vo2peak ) OR ( aerobic AND capacity ) OR ( an*robic AND threshold ) OR ( oxygen AND uptake ) ) )

141,648

Search Result 1033

Limit human 637

Limit English 499

**Ovid MEDLINE(R) ALL <1946 to July 27, 2024>**

1 wearable.mp. 32441

2 fitness tracker.mp. 232

3 pedometer.mp. 2522

4 (sensor or biosensor).mp. 184666

5 smartwatch*.mp. 1368

6 accelerometer*.mp. 20948

7 1 or 2 or 3 or 4 or 5 or 6 227013

8 (predict* or estimat* or calculat*).mp. 4386973

9 cardiorespiratory fitness.mp. 9154

10 (an*robic or lactate threshold).mp. 101035

11 (VO2 or VO2max* or VO2peak).mp. 30907

12 oxygen uptake.mp. 24022

13 aerobic capacity.mp. 6803

14 9 or 10 or 11 or 12 or 13 152165

15 7 and 8 and 14 664

16 limit 15 to english language 652

17 limit 16 to humans 450

**Embase <1996 to 2024 Week 30> 27 July 2024**

1 wearable.mp. 34621

2 fitness tracker.mp. 309

3 pedometer.mp. 4631

4 (sensor or biosensor).mp. 253621

5 smartwatch*.mp. 1635

6 accelerometer*.mp. 30404

7 1 or 2 or 3 or 4 or 5 or 6 302133

8 (predict* or estimat* or calculat*).mp. 5355741

9 cardiorespiratory fitness.mp. 13658

10 (an*robic or lactate threshold).mp. 102719

11 (VO2 or VO2max* or VO2peak).mp. 38210

12 oxygen uptake.mp. 27178

13 aerobic capacity.mp. 15496

14 9 or 10 or 11 or 12 or 13 164868

15 7 and 8 and 14 1087

16 limit 15 to english language 1079

17 limit 16 to humans 889
